# Antimicrobial activity of yeasts against some pathogenic bacteria

**DOI:** 10.14202/vetworld.2017.979-983

**Published:** 2017-08-24

**Authors:** Gamal Younis, Amal Awad, Rehab E. Dawod, Nehal E. Yousef

**Affiliations:** 1 Department of Bacteriology, Mycology and Immunology, Faculty of Veterinary Medicine, Mansoura University, 35516 Mansoura, Egypt; 2 Department of Bacteriology, Animal Health Research Institute, Damietta Branch, Damietta, Egypt

**Keywords:** antimicrobial, meat products, milk, pathogenic bacteria, yeasts

## Abstract

**Aim:**

This study was designed to isolate and identify yeast species from milk and meat products, and to test their antimicrobial activity against some bacterial species.

**Materials and Methods:**

A total of 160 milk and meat products samples were collected from random sellers and super markets in New Damietta city, Damietta, Egypt. Samples were subjected to yeast isolation procedures and tested for its antimicrobial activity against *Staphylococcus aureus*, *Pseudomonas aeruginosa*, and *Escherichia coli*. In addition, all yeast species isolates were subjected to polymerase chain reaction (PCR) for detection of *khs* (kievitone hydratase) and *pelA* (pectate degrading enzyme)genes.

**Results:**

The recovery rate of yeasts from sausage was 20% (2/10) followed by kareish cheese, processed cheese, and butter 10% (1/10) each as well as raw milk 9% (9/100), and fruit yoghurt 30% (6/20). Different yeast species were recovered, namely, *Candida kefyr* (5 isolates), *Saccharomyces cerevisiae* (4 isolates), *Candida intermedia* (3 isolates), *Candida tropicalis* (2 isolates), *Candida lusitaniae* (2 isolates), and *Candida krusei* (1 isolate). *khs* gene was detected in all *S. cerevisiae* isolates, however, *pelA* gene was not detected in all identified yeast species. Antimicrobial activity of recovered yeasts against the selected bacterial species showed high activity with *C. intermedia* against *S. aureus* and *E. coli*, *C. kefyr* against *E. coli*, and *C. lusitaniae* against *S. aureus*. Moderate activities were obtained with *C. tropicalis*, *C. lusitaniae*, and *S. cerevisiae* against *E. coli*; meanwhile, all the tested yeasts revealed a very low antimicrobial activity against *P. aeruginosa*.

**Conclusion:**

The obtained results confirmed that some kinds of yeasts have the ability to produce antimicrobial compounds that could inhibit some pathogenic and spoilage bacteria and these antimicrobial activity of yeasts enables them to be one of the novel agents in controlling spoilage of food.

## Introduction

Yeasts are one of the microorganisms found in milk and meat products. They are found in raw materials and in processed milk and meat products such as sausage, fruit yoghurt, and cheeses. Yeasts have the ability to produce antimicrobial compounds that could inhibit growth of harmful mold or bacteria [1]. In past two decades, there are few researchs conducted to investigate the role of naturally occurring yeasts for inhibiting the growth of foodborne bacteria with various mechanisms [2]. Chemical food preservatives are used commonly extending the shelf life and improving the safety of food by inhibiting the growth of pathogenic and spoilage bacteria. However, increasing fears of consumers about their toxicity and antimicrobial resistant pathogens that found in food, which constitute a direct risk to public health and this leads to searching for alternative methods of food preservation as biopreservation [3]. Biopreservation means the use of natural or controlled microorganisms, or their antimicrobial compounds, to prolong the shelf life of food and to improve the safety of food. The suitability of yeast biocontrol agents and their strategies to enhance stress tolerance are important to their efficacy and commercial application [4].

Some kinds of yeasts secrete toxins and these types called killer yeasts. These yeasts can inhibit growth of other yeast strains and also, have antimicrobial activities inhibiting growth of mold and bacteria [1]. Killer yeasts are found naturally in rotten vegetables and fruits and have inhibitrory effects on the growth of other microorganisms. In addition, the yeasts which showed killer activity during beer and wine production and preservation of food are able to combat harmful microorganisms [5].

*Saccharomyces cerevisiae* is one of the widely studied microorganisms, and it has been involved in many biotechnological processes due to its good fermentation capacity [6]. Furthermore, it has probiotic and health benefit that has been reported recently [7]. Inhibitory effects to *S. cerevisiae* killer strains were discovered in 1963 and related to secretion of some toxins from this time to now, k1 and k28 are most common toxins [8]. In addation to *S. cerevisiae*, production of killer toxins has been discussed among many species of yeasts including *Candida*, *Cryptococcus, Kluyveromyces, Debaromyces, Pichia, Williopsis*, and *Zygosaccharomyces* [9,10].

The main objectives of this study was to isolate and identify different species of yeasts from milk and meat products, and testing their inhibitory effects against some pathogenic bacterial species as well as detection of *khs* and *pelA* genes in the isolated yeast strains using PCR assay.

## Materials and Methods

### Ethical approval

There was no need of ethical approval in this study because no live animals were involved. Milk and meat product samples were collected as per standard sampling methods.

### Samples collection

A total of 160 samples including, 100 samples from raw milk, 50 samples from milk products including, fruit yoghurt (20), kareish cheese (10), processed cheese (10) and butter (10), and 10 processed meat products samples (sausage) were subjected to yeast isolation procedures. All samples were collected from random sellers and super markets located at New Damietta city, Damietta, Egypt. Samples were collected aseptically in sterile bags separately and were transmitted directly to laboratory.

### Preparation of samples and isolation of yeasts

One gram from each sample was inoculated in Glucose Yeast Extract Broth (GYEB) and incubated at 27°C for 24 h. A loopful from the previous inoculated broth was subcultured onto Sabouraud dextrose agar (Oxoid) plates and incubated for 48 h at 37°C [11]. Separate colony from each of suspected yeasts was picked up and streaked onto SDA plate, incubated at 27°C for 48 h to get a pure culture and stored at 4°C for further examination.

### Identification of yeast isolates

Yeasts were identified according to Wickerham and Burton [12] and Lodder and Kreger Van Rij [13] using traditional methods of identification including, growth on SDA plates, sugar fermentation, sugar assimilation, nitrate assimilation, ascospore formation, vegetative reproduction, and urea hydrolysis. Further identification of yeast isolates was done using VITEK 2 Compact method (BioMérieux, Marcy l’Étoile, France).

### Antimicrobial activity test

Antimicrobial activity of yeast isolates against bacterial species was done according to Roostita *et al*. [14]. Briefly, yeast colony from each isolated species was sub cultured into 15 ml GYEB and was incubated at 27°C for 48 h. From the incubated broth, a loopful was cultivated aseptically onto SDA plate, then, the inoculated plates were incubated at 37°C for 48 h. One isolate from each of *S. aureus*, *E. coli*, and *P. aeruginosa* (Department of Bacteriology, Mycology and Immunology, Faculty of Veterinary Medicine, Mansoura University, Egypt) was cultivated into 15 ml nutrient broth and was incubated at 37°C for 24 h, then, they were swept separately onto nutrient agar (NA) plates using sterile swabs. From the previously prepared yeast agar plates, few yeast colonies were carefully picked up using platinum loop and placed in the center of inoculated NA plates with *S. aureus*, *E. coli* and *P. aeruginosa*. Inoculated plates were incubated at 37°C for 24 h, then, clear inhibition zones were measured using ruler.

### Molecular detection of *khs* and *pelA* genes

Yeast isolates were subjected to PCR for detection of *khs* and *pelA* genes according to Suga *et al*. [15]. Yeast DNA was extracted using QIAamp DNeasy Plant Mini kit Catalogue No. 69104 (Qiagen, Germany). The primer pairs used (sequence, target gene, and PCR product) are listed in Table 1. PCR was performed in a total volume of 25 µL consisting of 12.5 µL of 2X PCR Master Mix (Emeraldamp GT PCR master mix [Takara, Japan] Code No. RR310A), 1 µL of each primer, and 6 µL DNA templates and the volume of the reaction mixture was completed to 25 µL using nuclease free water. PCR program for both *khs* and *pelA* genes was summarized in Table 2. The amplicons were analyzed by electrophoresis in 1.5% agarose gels (AB gene, USA) stained with ethidium bromide (Sigma, Germany) then photographed using a gel documentation system [16].

**Table-1:** Oligonucleotide primers sequences.

Gene	Sequence	Amplified product	Reference
*khs*	AAGCATCCGAAACAGTACT	919 bp	Suga *et al*., [33]
	TCAAGGATGCTGCTAAGCTG		
*pelA*	ATCGAATTCATGAAGTTCACTGCTGCTTTC	727 bp	
	ACGGAATTCGCAGCTCGTGGTGGAGCCAGT		

*khs*: Kievitone hydratase gene

**Table-2:** Cycling conditions of the different primers during PCR.

Target gene	Primary denaturation	No. of cycles	Secondary denaturation	Annealing temperature	Extension	Final extension
*khs*	95°C	35	94°C	53°C	72°C	72°C
	5 min		30 s	50 s	50 s	10 min
*pelA*	95°C	35	94°C	55°C	72°C	72°C
	5 min		30 s	45 s	45 s	10 min

PCR=Polymerase chain reaction, *khs*=Kievitone hydratase gene

## Results and Discussion

Milk acts as a good nutritional medium for yeasts growth; yeasts can ferment lactose, metabolize lactase, and produce carbonyl compounds, and volatile acids [17]. In the processed meat products, yeasts are objectionable, and they grow at a wide range of temperature and PH values, resulted in the spoilage of meat products. Their percent are used as an index of storage and hygienic safety of the product [18].

In this study, yeasts were isolated from 20% of examined sausage, 10% of kareish cheese, 10% of processed cheese, 10% of butter, 9% of raw milk, and 30% of fruit yoghurt (Table 3). These findings are lower than the findings of Ibrahim *et al.*, [19] who found that the occurrence of yeast species in the examined kareish cheese, Damietta processed cheese, and yoghurt samples were 100%, 100%, and 50%, respectively. In this study, the high prevalence rate of yeasts in fruit yogurt might attributed to that the yoghurt offer a good condition for yeasts growth [20]. In addition, fruits increase percentage of yeasts by about 3.3-3.4 times [21].

**Table-3:** Incidence of yeasts in raw milk, milk products, and sausage.

Type of sample	Number of examined samples (160)	Positive samples

		Number (%)
Raw milk	100	9 (9)
Fruit yoghurt	20	6 (30)
Kareish cheese	10	1 (10)
Processed cheese	10	1 (10)
Butter	10	1 (10)
Sausage	10	2 (20)

In the current study, many kinds of yeasts associated with milk and meat products were identified including, *C. krusei, C.kefyr (Kluyveromyces* *marxianus), C. tropicalis, C. intermedia, S. cerevisiae* from raw milk samples, *C. intermedia, C. lusitaniae* and *C. tropicalis* from fruit yoghurt, *C*. *lusitaniae* from kareish cheese, *C. kefyr* (*K. marxianus*) from butter and finally from sausage, *C. kefyr* (*K. marxianus*), and *S. cerevisiae* were recovered (Table 4). These findings are similar to the findings of many investigators [22-24] who identified nearly similar yeast species from milk and milk products.

**Table-4:** Different types of recovered yeasts from examined samples.

Type of yeast	Raw milk	Fruit yoghurt	Kareish cheese	Processed cheese	Butter	Sausage	Total

							Number (%)
*C. kefyr (K. marxianus)*	3	-	-	1	1	-	5 (25)
*C. krusei*	1	-	-	-	-	-	1 (5)
*C. tropicalis*	1	1	-	-	-	-	2 (10)
*C. lusitaniae*	-	1	1	-	-	-	2 (10)
*C. intermedia*	2	1	-	-	-	-	3 (15)
*S. cerevisiae*	2	-	-	-	-	2	4 (20)
Unidentified yeasts	-	3	-	-	-	-	3 (15)

C. kefyr=Candida kefyr, K. marxianus=Kluyveromyces marxianus, C. krusei=Candida krusei, C. tropicalis=Candida tropicalis, C. lusitaniae=Candida lusitaniae, C. intermedia=Candida intermedia, S. cerevisiae=Saccharomyces cerevisiae

In this study, some yeast isolates showed variable antimicrobial activities against *S. aureus, E. coli*, and *P. aeruginosa. C. intermedia* showed high antimicrobial activity against *E. coli* (20 mm) and *S. aureus* (24 mm) with large clear inhibition zone and low antimicrobial activity against *P. aeruginosa* (6 mm). *C. kefyr* showed high antimicrobial activity against *E. coli* (20 mm), low antimicrobial activity against *S. aureus* (8 mm), and no activity against *P. aeruginosa*. *C. tropicalis* showed moderate antimicrobial activity against *E. coli* (14 mm) and negative antimicrobial activity against *S. aureus* as well as *P. aeruginosa*. *C. lusitaniae* showed high antimicrobial activity against *S. aureus* (22 mm), moderate antimicrobial activity against *E. coli* (12 mm) and negative result against *P. aeruginosa*. *S. cerevisiae* showed moderate antimicrobial activity against *E. coli* (12 mm), low antimicrobial activity against *S. aureus* (8 mm) and negative antimicrobial activity against *P. aeruginosa* (5 mm) as shown in Table 5. These findings confirming the previous results of Roostita *et al*. [14], Abd Elatif *et al*. [25] who tested yeast species isolates recovered from livestock products on some pathogenic bacteria such as *P. aeruginosa, E. coli*, and *S. aureus*. Furthermore, Rajkowska *et al*. [26] also found a decreasing in *S. aureus* cells number after incubation with probiotic yeasts. The antimicrobial activity of the isolated yeasts against the used bacterial species might be attributed to production of some yeast products such as, volatile thermolabile toxic extract [27]. Another yeast products such as antilisterial hydrophobic peptides and mycocins against bacterial pathogens were also recently investigated [28-30].

**Table-5:** Antimicrobial activity of recovered yeasts against *E. coli*, *S. aureus*, and *P. aeruginosa*.

Yeast type	*E. coli*		*S. aureus*		*P. aeruginosa*	
		
	DIZ	Result	DIZ	Result	DIZ	Result
*C. intermedia*	20	+++	24	++++	6	+
*C. kefyr* *(K. marxianus)*	20	+++	8	+	-	-
*C. tropicalis*	14	++	-	-	-	-
*C. lusitaniae*	12	++	22	++++	-	-
*C. krusei*	-	-	-	-	-	-
*S. cerevisiae*	12	++	8	+	5	-

*E. coli*=*Escherichia coli*, *S. aureus*=*Staphylococcus aureus*, *P. aeruginosa*=*Pseudomonas aeruginosa*, DIZ=Diameter of inhibition zone by mm without zone of yeast growth. - (negative): 0-5 mm, + (low): 6-10 mm, ++ (moderate): 11-15 mm, +++ (high): 16-20 mm, ++++ (very high): 21-25 mm

**Table-6:** Results of PCR for detection of *khs* and *pelA* genes in different yeast isolates.

Samples types	Isolates	*khs* gene	*pelA* gene
Raw milk	*S. cerevisiae*	+	-
Raw milk	*C. tropicalis*	-	-
Raw milk	*C. intermedia*	-	-
Raw milk	*C. intermedia*	-	-
Raw milk	*S. cerevisiae*	+	-
Raw milk	*C. kefyr*	-	-
Raw milk	*C. kefyr*	-	-
Raw milk	*C. kefyr*	-	-
Raw milk	*C. krusei*	-	-
Fruit yoghurt	Unidentified yeasts	-	+
Fruit yoghurt	Unidentified yeasts		+
Fruit yoghurt	*C. intermedia*	-	-
Fruit yoghurt	*C. lusitaniae*	-	-
Fruit yoghurt	*Unidentified yeasts*	-	+
Fruit yoghurt	*C. tropicalis*	-	-
Sausage	*S. cerevisiae*	+	-
Sausage	*S. cerevisiae*	+	-
Kareish cheese	*C. lusitaniae*	-	-
Butter	*C. kefyr*	-	-
Processed cheese	*C. kefyr*	-	-

*C. kefyr=Candida kefyr*, *C. krusei*=*Candida krusei*, *C. tropicalis*=*Candida tropicalis*, *C. lusitaniae*=*Candida lusitaniae*, *C. intermedia*=*Candida intermedia*, *S. cerevisiae*=*Saccharomyces cerevisiae*, *Khs*=Kievitone hydratase gene, PCR=Polymerase chain reaction

**Table-7:** Cumulative results of PCR for detection of *khs* and *pelA* genes in different strains.

Yeast strain	Number of isolates	*Khs* gene	*PelA* gene
*S. cerevisiae*	4	+	-
*C. kefyr*	5	-	-
*C. intermedia*	3	-	-
*C. lusitaniae*	2	-	-
*C. tropicalis*	2	-	-
*C. krusei*	1	-	-
Unidentified yeasts	3	-	+

*C. kefyr*=*Candida kefyr*, *C. krusei*=*Candida krusei*, *C. tropicalis*=*Candida tropicalis*, *C. lusitaniae*=*Candida lusitaniae*, *C. intermedia*=*Candida intermedia*, *S. cerevisiae*=*Saccharomyces cerevisiae*, PCR=Polymerase chain reaction, *khs*=Kievitone hydratase gene

**Figure-1:**
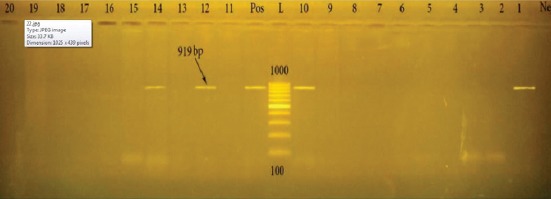
Agarose gel electrophoresis of polymerase chain reaction products on different yeast species demonstrating *khs* gene at 919 bp. Pos: Positive control, L: 100 bp DNA ladder, Neg: Negative control.

**Figure-2:**
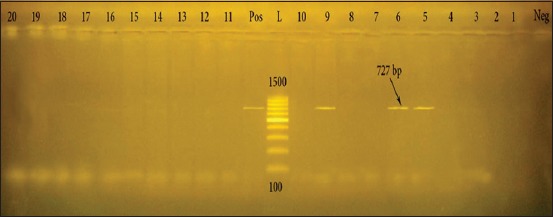
Agarose gel electrophoresis of polymerase chain reaction products on different yeast species demonstrating *pelA* gene at 727 bp. Pos: Positive control, L: 100 bp DNA ladder, Neg: Negative control.

Among the isolated yeast species, *S. cerevisiae* is considered a one of the killer yeasts that has *khs* gene as a killer mechanism [31]. In this study, *khs* gene was identified using PCR assay in *S. cerevisiae* which may be responsible for the bacterial growth inhibition on the inoculated plates (Figure-1). *khs* gene was previously identified in *Fusarium phaseoli* [15] and in *Nectria* *haematococca* [32]. In this study, *pel*A gene (pectate degrading enzyme) could not be identified in all identified yeast species (Tables 6 and 7), but it could be identified in three unidentified yeast species (Figure-2), *pel*A gene found in other fungal species including *Fusarium solani* spp. Pisi (MP VI), xanthoxyli (MP IV), batatas (MP II), and mori (MP III) [33,34].

## Conclusion

In this study, different kinds of yeasts had the ability to produce antimicrobial compounds that could inhibit the growth of the selected pathogenic bacterial species. *khs* gene which responsible for the killer mechanism in *S. cerevisiae* had been successfully identified in all recovered *S. cerevisiae* isolates.

## Authors’ Contributions

GY planned and design the study, shared in data analysis and revised the manuscript, NEY and AA performed the tests, wrote the manuscript, and analyzed the data. RED shared in performance of tests and wrote a part of manuscript. All authors read and approved the final manuscript.
